# 

**Published:** 1890-12

**Authors:** 


### INDEX TO VOLUME XI.

---

### ORIGINAL COMMUNICATIONS.

PAGE

A Delicate Operation tested by Time. By Henry N. Dodge, M.D., D.D.S., Morristown, New Jersey . . . . . . . . . . . . . . . . . 527
A New Method for the Treatment of Fractures of the Maxilla. By Edward H. Angle, D D.S., Minneapolis, Minnesota . . . . . . . . . . 330
An Interesting and Rare Case. By L. P. Blair, D.D.S. . . . . . . . . 529
An Interesting Operation. By George W. Warren . . . . . . . . . . 530
Bleaching of Discolored Dentine practically considered. By E. P. Wright, D.D.S., Richmond, Virginia . . . . . . . . . . . . . . . . . . . 70
Correcting Irregularities by the Spring of Gold Bands. By B. S. Byrnes, D.D.S., Memphis, Tennessee . . . . . . . . . . . . . . . . . . . 263
Dental Education. By C. Stoddard Smith, D.D.S. . . . . . . . . . . 339
Description of a Specimen of Cleft Palate. By Johnson Symington, M.D., F.R.S.E. . . . . . . . . . . . . . . . . . . . . . . . . 204
Fermentation: Its Cause and Effects. By Ernest Laplace, A.M., M.D., Philadelphia . . . . . . . . . . . . . . . . . . . . . . . . 1
Formations in the Pulp-Cavity. By Wm. P. Cooke, D.M.D. . . . . . 725
Fracture of the Jaws. By Dr. D. W. Fellows, Portland, Maine . . . . 77
From Another Stand-Point. By Dr. J. Allen Osman, Newark, N. J. . . 739
Gasoline Blow-Pipe. By J. J. Reed, Kansas . . . . . . . . . . . . . . 675
How to splice Engine Bands. By George A. Maxfield, D.D.S., Holyoke, Massachusetts . . . . . . . . . . . . . . . . . . . . . . . . 75
Improved Dental Ledger and Book-Keeping. By Dr. L. C. Bryan, Basel, Switzerland . . . . . . . . . . . . . . . . . . . . . . . . 606
Limitations in Treatment and Filling of Roots. By Dr. J. A. Bazin, Montreal, Canada . . . . . . . . . . . . . . . . . . . . . . . 522
Local Anæsthesia by Nitrous Oxide: A Convenient Method of applying it. By G. L. Curtis, M.D., D.D.S., Syracuse, New York . . . . . . 403
Matrix or Ring Fillings. By Dr. L. C. Bryan, Basel, Switzerland . . . 661
New and Old German Bridge-Work. By Dr. L. C. Bryan, Basel, Switzerland . . . . . . . . . . . . . . . . . . . . . . . . . . 460

PAGE
Observations on Preparation, Discrimination, Prevention, Reparation, Association, and Publication as applied to Dentistry. By Charles B. Atkinson, D.D.S. . . . . . . . . . . . . . . . . . . . . . . . 591
On the Dangers arising from Syphilis in the Practice of Dentistry. By L. Duncan Bulkley, A.M., M.D. . . . . . . . . . . . . . . . . 449, 513
Oral Deformities and their Correction. By Kasson C. Gibson, New York City . . . . . . . . . . . . . . . . . . . . . . . . . . . . 385
Oral Surgery Clinic. By Professor John S. Marshall, M.D. . . . . 139, 337
Rapid Method of inserting and finishing Contour Fillings without the Aid of a Matrix. By J. E. Waitt, D.M.D. . . . . . . . . . . . . 136
Reflex Ocular and Facial Symptoms of Nasal Disease. By Edward S. Peck, A.M., M.D. . . . . . . . . . . . . . . . . . . . . . . . 321
Sensation (concluded). By George W. Whitefield, M.D., D.D.S., Chicago, Illinois . . . . . . . . . . . . . . . . . . . . . . . . . 16
Some Methods of making and retaining Removable Appliances for correcting Irregularities of the Teeth. By V. H. Jackson, D.D.S., New York . . . . . . . . . . . . . . . . . . . . . . . . . . . 199
Some Practical Points learned at Society Meetings and Clinics. By B. A. R. Ottolengui, New York City . . . . . . . . . . . . . . . . . 267
Some Thoughts on Crown- and Bridge-Work. By W. Mitchell, D.D.S., London, England . . . . . . . . . . . . . . . . . . . . . . . . 462
Tannic Acid: Its Internal Administration for Hemorrhage after Tooth Extraction. By Dr. W. L. Roberts, Weymouth, Massachusetts . . . 457
The Combination of Tin and Gold as a Filling-Material. By Dr. H. F. Hamilton . . . . . . . . . . . . . . . . . . . . . . . . . . . 738
The Dental Crown and its Enabling Method of Removable Bridge-Work. By William H. Gates, D.D.S., Philadelphia . . . . . . . . . . . . 257
The Herbst Method of filling with Glass. By James A. Bruce, D.D.S., Melbourne, Australia . . . . . . . . . . . . . . . . . . . . . . 676
The Massage Treatment in Dental Pathological Conditions. By W. F. Rehfuss, D.D.S. . . . . . . . . . . . . . . . . . . . . . . . . 581
The Relations of Dentistry to Medicine, and Aseptic Dentistry. By J. S. Wight, M.D. . . . . . . . . . . . . . . . . . . . . . . . . . 129
The Relations of the Tooth-Pulp to other Tooth Tissues. By George H. McCausey, Janesville, Wisconsin . . . . . . . . . . . . . . . . . 193
The Treatment of Proximate Cavities. By George Howe Winkler, M.D., D.D.S. . . . . . . . . . . . . . . . . . . . . . . . . . . . . 729
The Treatment of Proximate Surfaces. By George A. Wilson, D.D.S. . 732
The Use of a Matrix in contouring Fillings. By Dr. H. A. Baker . . . 608
The Waste Products of the Body. By Professor Charles Mayr, Springfield, Massachusetts . . . . . . . . . . . . . . . . . . . . . . . 9
Tomato-Poisoning. By William A. Mills, D.D.S., Baltimore, Maryland 209
Treatment of the Teeth during Pregnancy. By W. H. Dwinelle, M.D., D.D.S., New York City . . . . . . . . . . . . . . . . . . . . . . 65
Various Modes adopted in filling Teeth. By Wilhelm Sachs, D.D.S., Breslau, Germany . . . . . . . . . . . . . . . . . . . . . . . . 600
What is the Essential Basis of Professional Ethics, and the Proper Relations of Trade? By J. Morgan Howe, M.D., D.D.S. . . . . . . . 664

### REPORTS OF SOCIETY MEETINGS.

PAGE
American Academy of Dental Science . . . . . 115, 141, 274, 499, 636, 782
American Dental Association . . . . . . . . . . . . . . . 531, 611, 678, 746
American Dental Society of Europe . . . . . . . . . . . 238, 473, 643, 764
Chicago Dental Club . . . . . . . . . . . . . . . . . . . . . . . . 348
Congress of the Italian Odontological Society . . . . . . . . . . . . 235
Massachusetts State Dental Society . . . . . . . . . . . . . . . . . 27
National Association of Dental Examiners . . . . . . . . . . . . . . 567
National Association of Dental Faculties . . . . . . . . . . . . . . 565
New Jersey State Dental Society . . . . . . . . . . . . . . . . . 41, 102, 772
New York Odontological Society . . . . 211, 362, 420, 468, 552, 618, 686, 755
Odontological Society of Pennsylvania . . . . . . . . . . . . 49, 81, 296, 433
Seventh Annual Session of the Maryland State Dental Association,
168, 222, 286, 408, 491, 540, 645
Union Meeting, Springfield, Massachusetts . . . . . . 148, 229, 301, 481, 539

### EDITORIALS.

About Correct . . . . . . . . . . . . . . . . . . . . . . . . . . 122
A Call for an International Dental Congress . . . . . . . . . . . . . . 185
American Dental Association . . . . . . . . . . . . . . . . . . . . 184, 571
Au Revoir . . . . . . . . . . . . . . . . . . . . . . . . . . . . . 436
Deaths from Chloroform in New South Wales . . . . . . . . . . . . . 704
Dental Hygiene . . . . . . . . . . . . . . . . . . . . . . . . . . 246
Dental Societies . . . . . . . . . . . . . . . . . . . . . . . . . 310
Dr. J. N. Farrar's Work on Irregularities . . . . . . . . . . . . . . 703
National Association of Dental Examiners . . . . . . . . . . . . . . 574
National Association of Dental Faculties . . . . . . . . . . . . . . 573
Patent-Office Examiners . . . . . . . . . . . . . . . . . . . . . . . 53
Personal . . . . . . . . . . . . . . . . . . . . . . . . . . . . . 505
President Foster's Address . . . . . . . . . . . . . . . . . . . . . 652
Professor Miller's "Micro-Organisms of the Human Mouth" . . . . . . 789
Professor W. B. Miller . . . . . . . . . . . . . . . . . . . . . . . 701
Reflections on the Passing Year . . . . . . . . . . . . . . . . . . 788
Southern Dental Journal . . . . . . . . . . . . . . . . . . . . . . 247
Society Meetings . . . . . . . . . . . . . . . . . . . . . . . . . 378
Special Announcement . . . . . . . . . . . . . . . . . . . . . . . 436
Temporary Enlargement . . . . . . . . . . . . . . . . . . . . . . 655
That "Memorial Meeting" . . . . . . . . . . . . . . . . . . . . . . 56
The Conventions . . . . . . . . . . . . . . . . . . . . . . . . . 438
The National Association of Dental Faculties . . . . . . . . . . . . 182
The Need of the Hour . . . . . . . . . . . . . . . . . . . . . . . 506
The Present Need of the Journal . . . . . . . . . . . . . . . . . . 703
The Tenth International Medical Congress . . . . . . . . . . . . . . 123
The Union Dental Convention . . . . . . . . . . . . . . . . . . . . 508
Unsatisfactory Laws . . . . . . . . . . . . . . . . . . . . . . . . 120

### BIBLIOGRAPHY.

PAGE
BIBLIOGRAPHY . . . . . . . . . . . . . . . . . . . . . 248, 313, 439, 575, 790

### FOREIGN CORRESPONDENCE.

FOREIGN CORRESPONDENCE . . . . . . . . . . 57, 124, 186, 249, 314, 380, 441

### DOMESTIC CORRESPONDENCE.

DOMESTIC CORRESPONDENCE,
60, 127, 188, 253, 315, 383, 442, 509, 577, 655, 704

### OBITUARY.

OBITUARY . . . . . . . . . . . . . . . . . . . . . . . . . . . . . . . 658, 706

### CURRENT NEWS.

CURRENT NEWS . . . . 63, 128, 191, 255, 318, 384, 447, 511, 580, 659, 707, 799

**SUBSCRIBE FOR THE**

### International Dental Journal

### FOR 1890.

ITS FEARLESS, INDEPENDENT COURSE,
IN DEFENCE OF PROFESSIONAL INTERESTS, SHOULD MAKE
IT INDISPENSABLE TO EVERY DENTIST.

***ALL DEALERS ARE OUR AUTHORIZED AGENTS.***

Terms, $2.50 per Annum, in Advance.

---

### IDEAL BRUSHES

### The Prophylactic for Natural Teeth.

FIVE SIZES. FIVE GRADES BRISTLES.

### The Florence Dental Plate for Artificial Teeth,

A NECESSITY, A BLESSING.

### APPEAL TO EVERY DENTIST.

**DESCRIPTIVE CIRCULARS ON APPLICATION.**

### Florence Manufacturing Co.,

4-7-00-½.

Florence Mass.

SUBSCRIBE FOR THE

### International Dental Journal

**The Organ of the Profession,**

PUBLISHED BY THE

### INTERNATIONAL DENTAL PUBLICATION COMPANY:

**LOUIS JACK,** *President*, Philadelphia.
**BENJAMIN LORD,** *Vice President*, New York.
**C. N. PEIRCE,** *Treasurer*, Philadelphia.
**GEO. S. ALLAN,** *Secretary*, 51 W. 37th St., New York.

**W. XAVIER SUDDUTH, M.D., D.D.S., F.R.M.S.**

**Editor and Business Manager,**

**716 Filbert Street, Philadelphia, Pa.**

### FOREIGN CORRESPONDENTS:

Dr. ARKÖVY, Budapesth, Hungary.
W. GEO. BEERS, D.D.S., Ed. Dominion Dental Journal, Montreal.
L. C. BRYAN, D.D.S., Basel.
ALFRED BURNE, D.D.S., Sydney, New South Wales.
F. BUSCH, M.D., Berlin.
GEORGE CUNNINGHAM, Cambridge, Eng.
I. B. DAVENPORT, D.D.S., Paris.
PAUL DUBOIS, Ed. L'Odontologie, Paris.
WILLIAM DUNN, Jr., L.D.S., Florence.
A. H. EDWARDS, D.D.S., Madrid.
W. ST. GEORGE ELLIOTT, M.D., D.D.S., Lon.
OSWALD FERGUS, D.D.S., Glasgow, Scotland.
ALPHONSE FEUCHEL, Zahnarzt, St. Petersburg.
C. R. FLETCHER, D.D.S., Sao Paulo, Brazil.
WALTER HARRISON, L.D.S., D.M.D., Brighton, Eng.
ROBERT S. IVY, D.D.S., Shanghai, China.
N. S. JENKINS, D.D.S., Dresden.
F. KLAPPROTH, Zahnarzt, St. Petersburg.
L. KOLBE, " " "
Dr. GEO. P. E. KUHN, Paris.
W. BOWMAN MACLEOD, L.D.S., Edinburgh.
W. D. MILLER, Ph. D., M.D., D.D.S., Berlin.
WILLIAM SACHS, D.D.S., Breslau.
J. G. VAN MARTER, B.A., D.D.S., Rome.
J. M. WHITNEY, M.D., D.D.S., Honnolula.
LUDWIG WARNEKROS, Zahnarzt, Berlin.
LUDW. ADOLPH WEIL, M.D., Munich.
J. COWAN WOODBURN, M.D., Glasgow.

### STOCKHOLDERS:

Frank Abbott,
Geo. S. Allan,
W. W. Allport,
R. R. Andrews,
H. A. Baker,
A. E. Baldwin,
W. C. Barrett,
A. P. Beale,
S. T. Beale, Jr.,
C. S. Beck,
G. V. Black,
C. F. W. Bodecker,
E. A. Bogue,
W. G. A. Bonwill,
G. A. Brackett,
Thomas Bradley,
Edward C. Briggs,
Albert H. Brockway,
A. E. Brown,
Wm. Carr,
Geo. H. Chance,
G. F. Cheney,
Dwight M. Clapp,
John D. Codman,
Chas. D. Cook,
J. N. Crouse,
Edw. T. Darby,
H. S. Draper,
Wm. H. Dwinelle,
Chas. J. Essig,
J. N. Farrar,
T. Fillebrown,
W. B Finney,
T. A. Fletcher,
M. W. Foster,
Chas. E. Francis,
W. F. Fundenberg,
W. H. Fundenberg,
E. C. Fundenberg,
E. S. Gaylord,
J. P. Geran,
A. H. Gilson,
S. H. Guilford,
John I. Hart,
O. E. Hill,
J. F. P. Hodson,
J. Morgan Howe,
Robert Huey,
A. O. Hunt,
Louis Jack,
V. H. Jackson,
C. R. Jefferis,
C. A. Kingsbury,
Edw. C. Kirk,
C. R. E. Koch,
W. T. La Roche,
W. K. Leech,
F. A. Levy,
Wilbur F. Litch,
J. Bond Littig,
Benjamin Lord,
Geo. W. Lovejoy (Canada),
John S. Marshall,
H. J. McKellops,
Charles McManus,
James McManus,
Dan'l Neal McQuillan,
Horatio C. Meriam,
W. D. Miller (Berlin),
W. E. Page,
S. B. Palmer,
C. B. Parker,
D. D. Peabody,
C. N. Peirce,
S. G. Perry,
Chas. E. Pike,
W. A. Phreaner,
W. E. Pinkham,
W. H. Potter,
Chas. P. Pruyn,
H. C. Register,
Z. T. Sailer,
L. D. Shepard,
Eugene H. Smith,
B Holly Smith,
H. A. Smith,
S. G. Stevens,
W. X. Sudduth,
Eugene S. Talbott,
Ambler Tees,
J. D. Thomas,
James Truman,
Wm. H. Trueman,
W. W. Walker,
I. F. Wardwell,
G. W. Warren,
T. S. Waters,
Geo. W. Weld,
Julius G. W. Werner,
Jacob L. Williams,
R. B. Winder,
C. A. Woodward,
J. A. Woodward,
W. J. Younger.

SUBSCRIBE FOR THE

### International Dental Journal

**The Organ of the Profession,**

PUBLISHED BY THE

INTERNATIONAL DENTAL PUBLICATION COMPANY:

**LOUIS JACK,** *President,* Philadelphia.
**BENJAMIN LORD,** *Vice-President,* New York.
**C. N. PEIRCE,** *Treasurer,* Philadelphia.
**GEO. S. ALLAN,** *Secretary,* 51 W. 37th St., New York.

**W. XAVIER SUDDUTH, M.D., D.D.S., F.R.M.S.**

Editor and Business Manager,

716 Filbert Street, Philadelphia, Pa.

**FOREIGN CORRESPONDENTS:**

Dr. ARKÖVY, Budapesth, Hungary.
W. GEO. BEERS, D.D.S., Ed. Dominion Dental Journal, Montreal.
L. C. BRYAN, D.D.S., Basel.
ALFRED BURNE, D.D.S., Sydney, New South Wales.
F. BUSCH, M.D., Berlin.
GEORGE CUNNINGHAM, Cambridge, Eng.
I. B. DAVENPORT, D.D.S., Paris.
PAUL DUBOIS, Ed. L'Odontologie, Paris.
WILLIAM DUNN, Jr., L.D.S., Florence.
A. H. EDWARDS, D.D.S., Madrid.
W. ST. GEORGE ELLIOTT, M.D., D.D.S., Lon.
OSWALD FERGUS, D.D.S., Glasgow, Scotland.
ALPHONSE FEUCHEL, Zahnarzt, St. Petersburg.
C. R. FLETCHER, D.D.S., Sao Paulo, Brazil.
WALTER HARRISON, L.D.S., D.M.D., Brighton, Eng.
ROBERT S. IVY, D.D.S., Shanghai, China.
N. S. JENKINS, D.D.S., Dresden.
F. KLAPPROTH, Zahnarzt, St. Petersburg.
L. KOLBE, " " "
Dr. GEO. P. E. KUHN, Paris.
W. BOWMAN MACLEOD, L.D.S., Edinburgh.
W. D. MILLER, Ph. D., M.D., D.D.S., Berlin.
WILLIAM SACHS, D.D.S., Breslau.
J. G. VAN MARTER, B.A., D.D.S., Rome.
J. M. WHITNEY, M.D., D.D.S., Honnolula.
LUDWIG WARNEKROS, Zahnarzt, Berlin.
LUDW. ADOLPH WEIL, M.D., Munich.
J. COWAN WOODBURN, M.D., Glasgow.

STOCKHOLDERS:

Frank Abbott, Geo. S. Allan, W. W. Allport, R. R. Andrews, H. A. Baker, A. E. Baldwin, W. C. Barrett, A. P. Beale, S. T. Beale, Jr., C. S. Beck, G. V. Black, C. F. W. Bodecker, E. A. Bogue, W. G. A. Bonwill, C. A. Brackett, Thomas Bradley, Edward C. Briggs, Albert H. Brockway, A. E. Brown, Wm. Carr, Geo. H. Chance, G. F. Cheney, Dwight M. Clapp, John D. Codman, Chas. D. Cook, J. N. Crouse,
Edw. T. Darby, H. S. Draper, Wm. H. Dwinelle, Chas. J. Essig, J. N. Farrar, T. Fillebrown, W. B. Finney, T. A. Fletcher, M. W. Foster, Chas. E. Francis, W. F. Fundenberg, W. H. Fundenberg, E. C. Fundenberg, E. S. Gaylord, J. P. Geran, A. H. Gilson, S. H. Guilford, John I. Hart, O. E. Hill, J. F. P. Hodson, J. Morgan Howe, Robert Huey, A. O. Hunt, Louis Jack, V. H. Jackson, C. R. Jefferis,
C. A. Kingsbury, Edw. C. Kirk, C. R. E. Koch, W. T. La Roche, W. K. Leech, F. A. Levy, Wilbur F. Litch, J. Bond Littig, Benjamin Lord, Geo. W. Lovejoy (Canada), John S. Marshall, H. J. McKellops, Charles McManus, James McManus, Dan'l Neal McQuillan, Horatio C. Meriam, W. D. Miller (Berlin), W. E. Page, S. B. Palmer, C. B. Parker, D. D. Peabody, C. N. Peirce, S. G. Perry, Chas. E. Pike, W. A. Phreaner, W. E. Pinkham,
W. H. Potter, Chas. P. Pruyn, H. C. Register, Z. T. Sailer, L. D. Shepard, Eugene H. Smith, B. Holly Smith, H. A. Smith, S. G. Stevens, W. X. Sudduth, Eugene S. Talbott, Ambler Tees, J. D. Thomas, James Truman, Wm. H. Trueman, W. W. Walker, I. F. Wardwell, G. W. Warren, T. S. Waters, Geo. W. Weld, Julius G. W. Werner, Jacob L. Williams, R. B. Winder, C. A. Woodward, J. A. Woodward, W. J. Younger.

SUBSCRIBE FOR THE

### International Dental Journal

**The Organ of the Profession,**

PUBLISHED BY THE

### INTERNATIONAL DENTAL PUBLICATION COMPANY:

**LOUIS JACK,** *President*, Philadelphia.
**BENJAMIN LORD,** *Vice President*, New York.
**C. N. PEIRCE,** *Treasurer*, Philadelphia.
**GEO. S. ALLAN,** *Secretary*, 51 W. 37th St., New York.

**W. XAVIER SUDDUTH, M.D., D.D.S., F.R.M.S.**

Editor and Business Manager,

716 Filbert Street, Philadelphia, Pa.

**FOREIGN CORRESPONDENTS:**

Dr. ARKÖVY, Budapesth, Hungary.
W. GEO. BEERS, D.D.S., Ed. Dominion Dental Journal, Montreal.
L. C. BRYAN, D.D.S., Basel.
ALFRED BURNE, D.D.S., Sydney, New South Wales.
F. BUSCH, M.D., Berlin.
GEORGE CUNNINGHAM, Cambridge, Eng.
I. B. DAVENPORT, D.D.S., Paris.
PAUL DUBOIS, Ed. L'Odontologie, Paris.
WILLIAM DUNN, Jr., L.D.S., Florence.
A. H. EDWARDS, D.D.S., Madrid.
W. ST. GEORGE ELLIOTT, M.D., D.D.S., Lon.
OSWALD FERGUS, D.D.S., Glasgow, Scotland.
ALPHONSE FEUCHEL, Zahnarzt, St. Petersburg.
C. R. FLETCHER, D.D.S., Sao Paulo, Brazil.
WALTER HARRISON, L.D.S., D.M.D., Brighton, Eng.
ROBERT S. IVY, D.D.S., Shanghai, China.
N. S. JENKINS, D.D.S., Dresden.
F. KLAPPROTH, Zahnarzt, St. Petersburg.
L. KOLBE, " " "
Dr. GEO. P. E. KUHN, Paris.
W. BOWMAN MACLEOD, L.D.S., Edinburgh.
W. D. MILLER, Ph. D., M.D., D.D.S., Berlin.
WILLIAM SACHS, D.D.S., Breslau.
J. G. VAN MARTER, B.A., D.D.S., Rome.
J. M. WHITNEY, M.D., D.D.S., Honnolula.
LUDWIG WARNEKROS, Zahnarzt, Berlin.
LUDW. ADOLPH WEIL, M.D., Munich.
J. COWAN WOODBURN, M.D., Glasgow.

STOCKHOLDERS:

Frank Abbott,
Geo. S. Allan,
W. W. Allport,
R. R. Andrews,
H. A. Baker,
A. E. Baldwin,
W. C. Barrett,
A. P. Beale,
S. T. Beale, Jr.,
C. S. Beck,
G. V. Black,
C. F. W. Bodecker,
E. A. Bogue,
W. G. A. Bonwill,
C. A. Brackett,
Thomas Bradley,
Edward C. Briggs,
Albert H. Brockway,
A. E. Brown,
Wm. Carr,
Geo. H. Chance,
G. F. Cheney,
Dwight M. Clapp,
John D. Codman,
Chas. D. Cook,
J. N. Crouse,
Edw. T. Darby,
H. S. Draper,
Wm. H. Dwinelle,
A. R. Eaton,
Chas. J. Essig,
J. N. Farrar,
T. Fillebrown,
W. B. Finney,
T. A. Fletcher,
M. W. Foster,
Chas. E. Francis,
W. F. Fundenberg,
W. H. Fundenberg,
E. C. Fundenberg,
E. S. Gaylord,
J. P. Geran,
A. H. Gilson,
S. H. Guilford,
John I. Hart,
O. E. Hill,
J. F. P. Hodson,
J. Morgan Howe,
Robert Huey,
A. O. Hunt,
Louis Jack,
V. H. Jackson,
C. R. Jefferis,
C. A. Kingsbury,
Edw. C. Kirk,
C. R. E. Koch,
W. T. La Roche,
W. K. Leech,
F. A. Levy,
Wilbur F. Litch,
J. Bond Littig,
Benjamin Lord,
Geo. W. Lovejoy (Canada),
John S. Marshall,
H. J. McKellops,
Charles McManus,
James McManus,
Dan'l Neal McQuillan,
Horatio C. Meriam,
W. D. Miller (Berlin),
W. E. Page,
S. B. Palmer,
C. B. Parker,
D. D. Peabody,
C. N. Peirce,
S. G. Perry,
Chas. E. Pike,
W. A. Phreaner,
W. E. Pinkham,
W. H. Potter,
Chas. P. Pruyn,
H. C. Register,
Z. T. Sailer,
L. D. Shepard,
Eugene H. Smith,
B. Holly Smith,
H. A. Smith,
S. G. Stevens,
W. X. Sudduth,
Eugene S. Talbott,
Ambler Tees,
J. D. Thomas,
James Truman,
Wm. H. Trueman,
W. W. Walker,
I. F. Wardwell,
G. W. Warren,
T. S. Waters,
Geo. W. Weld,
Julius G. W. Werner,
Jacob L. Williams,
R. B. Winder,
C. A. Woodward,
J. A. Woodward,
W. J. Younger.

SUBSCRIBE FOR THE

### International Dental Journal

**The Organ of the Profession,**

PUBLISHED BY THE

### INTERNATIONAL DENTAL PUBLICATION COMPANY:

**LOUIS JACK,** *President,* Philadelphia.
**BENJAMIN LORD,** *Vice-President,* New York.
**C. N. PEIRCE,** *Treasurer,* Philadelphia.
**GEO. S. ALLAN,** *Secretary,* 51 W. 37th St., New York.

### W. XAVIER SUDDUTH, M.D., D.D.S., F.R.M.S.

**Editor and Business Manager,**

**716 Filbert Street, Philadelphia, Pa.**

### FOREIGN CORRESPONDENTS:

Dr. ARKÖVY, Budapesth, Hungary.
W. GEO. BEERS, D.D.S., Ed. Dominion Dental Journal, Montreal.
L. C. BRYAN, D.D.S., Basel.
ALFRED BURNE, D.D.S., Sydney, New South Wales.
F. BUSCH, M.D., Berlin.
GEORGE CUNNINGHAM, Cambridge, Eng.
I. B. DAVENPORT, D.D.S., Paris.
PAUL DUBOIS, Ed. L'Odontologie, Paris.
WILLIAM DUNN, Jr., L.D.S., Florence.
A. H. EDWARDS, D.D.S., Madrid.
W. ST. GEORGE ELLIOTT, M.D., D.D.S., Lon.
OSWALD FERGUS, D.D.S., Glasgow, Scotland.
ALPHONSE FEUCHEL, Zahnarzt, St. Petersburg.
C. R. FLETCHER, D.D.S., Sao Paulo, Brazil.
WALTER HARRISON, L.D.S., D.M.D., Brighton, Eng.
ROBERT S. IVY, D.D.S., Shanghai, China.
N. S. JENKINS, D.D.S., Dresden.
F. KLAPPROTH, Zahnarzt, St. Petersburg.
L. KOLBE, " " "
Dr. GEO. P. E. KUHN, Paris.
W. BOWMAN MACLEOD, L.D.S., Edinburgh.
W. D. MILLER, Ph. D., M.D., D.D.S., Berlin.
WILLIAM SACHS, D.D.S., Breslau.
J. G. VAN MARTER, B.A., D.D.S., Rome.
J. M. WHITNEY, M.D., D.D.S., Honnolulu.
LUDWIG WARNEKROS, Zahnarzt, Berlin.
LUDW. ADOLPH WEIL, M.D., Munich.
J. COWAN WOODBURN, M.D., Glasgow.

### STOCKHOLDERS:

Frank Abbott,
Geo. S. Allan,
W. W. Allport,
R. R. Andrews,
H. A. Baker,
A. E. Baldwin,
W. C. Barrett,
A. P. Beale,
S. T. Beale, Jr.,
C. S. Beck,
G. V. Black,
C. F. W. Bodecker,
E. A. Bogue,
W. G. A. Bonwill,
C. A. Brackett,
Thomas Bradley,
Edward C. Briggs,
Albert H. Brockway,
A. E. Brown,
Wm. Carr,
Geo. H. Chance,
G. F. Cheney,
Dwight M. Clapp,
John D. Codman,
Chas. D. Cook,
J. N. Crouse,
Edw. T. Darby,
H. S. Draper,
Wm. H. Dwinelle,
A. R. Eaton,
Chas. J. Essig,
J. N. Farrar,
T. Fillebrown,
W. B. Finney,
T. A. Fletcher,
M. W. Foster,
Chas. E. Francis,
W. F. Fundenberg,
W. H. Fundenberg,
E. C. Fundenberg,
E. S. Gaylord,
J. P. Geran,
A. H. Gilson,
S. H. Guilford,
John I. Hart,
O. E. Hill,
J. F. P. Hodson,
J. Morgan Howe,
Robert Huey,
A. O. Hunt,
Louis Jack,
V. H. Jackson,
C. R. Jefferis,
C. A. Kingsbury,
Edw. C. Kirk,
C. R. E. Koch,
W. T. La Roche,
W. K. Leech,
F. A. Levy,
Wilbur F. Litch,
J. Bond Littig,
Benjamin Lord,
Geo. W. Lovejoy (Canada),
John S. Marshall,
H. J. McKellops,
Charles McManus,
James McManus,
Dan'l Neal McQuillan,
Horatio C. Meriam,
W. D. Miller (Berlin),
W. E. Page,
S. B. Palmer,
C. B. Parker,
D. D. Peabody,
C. N. Peirce,
S. G. Perry,
Chas. E. Pike,
W. A. Phreaner,
W. E. Pinkham,
W. H. Potter,
Chas. P. Pruyn,
H. C. Register,
Z. T. Sailer,
L. D. Shepard,
Eugene H. Smith,
B. Holly Smith,
H. A. Smith,
S. G. Stevens,
W. X. Sudduth,
Eugene S. Talbott,
Ambler Tees,
J. D. Thomas,
James Truman,
Wm. H. Trueman,
W. W. Walker,
I. F. Wardwell,
G. W. Warren,
T. S. Waters,
Geo. W. Weld,
Julius G. W. Werner,
Jacob L. Williams,
R. B. Winder,
C. A. Woodward,
J. A. Woodward,
W. J. Younger.

SUBSCRIBE FOR THE

### International Dental Journal

**The Organ of the Profession,**

PUBLISHED BY THE

### INTERNATIONAL DENTAL PUBLICATION COMPANY:

**LOUIS JACK,** *President,* Philadelphia.

**BENJAMIN LORD,** *Vice-President,* New York.

**C. N. PEIRCE,** *Treasurer,* Philadelphia.

**GEO. S. ALLAN,** *Secretary,* 51 W. 37th St., New York.

---

**W. XAVIER SUDDUTH, M.D., D.D.S., F.R.M.S.**

Editor and Business Manager,

716 Filbert Street, Philadelphia, Pa.

---

**FOREIGN CORRESPONDENTS:**

Dr. ARKÖVY, Budapesth, Hungary.
W. GEO. BEERS, D.D.S., Ed. Dominion Dental Journal, Montreal.
L. C. BRYAN, D.D.S., Basel.
ALFRED BURNE, D.D.S., Sydney, New South Wales.
F. BUSCH, M.D., Berlin.
GEORGE CUNNINGHAM, Cambridge, Eng.
I. B. DAVENPORT, D.D.S., Paris.
PAUL DUBOIS, Ed. L'Odontologie, Paris.
WILLIAM DUNN, Jr., L.D.S., Florence.
A. H. EDWARDS, D.D.S., Madrid.
W. ST. GEORGE ELLIOTT, M.D., D.D.S., Lon.
OSWALD FERGUS, D.D.S., Glasgow, Scotland.
ALPHONSE FEUCHEL, Zahnarzt, St. Petersburg.
C. R. FLETCHER, D.D.S., Sao Paulo, Brazil.
WALTER HARRISON, L.D.S., D.M.D., Brighton, Eng.
ROBERT S. IVY, D.D.S., Shanghai, China.
N. S. JENKINS, D.D.S., Dresden.
F. KLAPPROTH, Zahnarzt, St. Petersburg.
L. KOLBE, " " "
Dr. GEO. P. E. KUHN, Paris.
W. BOWMAN MACLEOD, L.D.S., Edinburgh.
W. D. MILLER, Ph. D., M.D., D.D.S., Berlin.
WILLIAM SACHS, D.D.S., Breslau.
J. G. VAN MARTER, B.A., D.D.S., Rome.
J. M. WHITNEY, M.D., D.D.S., Honnolula.
LUDWIG WARNEKROS, Zahnarzt, Berlin.
LUDW. ADOLPH WEIL, M.D., Munich.
J. COWAN WOODBURN, M.D., Glasgow.

---

STOCKHOLDERS:

Frank Abbott,
Geo. S. Allan,
W. W. Allport,
R. R. Andrews,
H. A. Baker,
A. E. Baldwin,
W. C. Barrett,
A. P. Beale,
S. T. Beale, Jr.,
C. S. Beck,
G. V. Black,
C. F. W. Bodecker,
E. A. Bogue,
W. G. A. Bonwill,
C. A. Brackett,
Thomas Bradley,
Edward C. Briggs,
Albert H. Brockway,
A. E. Brown,
Wm. Carr,
Geo. H. Chance,
G. F. Cheney,
Dwight M. Clapp,
John D. Codman,
Chas. D. Cook,
J. N. Crouse,
Edw. T. Darby,
H. S. Draper,
Wm. H. Dwinelle,
A. R. Eaton,
Chas. J. Essig,
J. N. Farrar,
T. Fillebrown,
W. B Finney,
T. A. Fletcher,
M. W. Foster,
Chas. E. Francis,
W. F. Fundenberg,
W. H. Fundenberg,
E. C. Fundenberg,
E. S. Gaylord,
J. P. Geran,
A. H. Gilson,
S. H. Guilford,
John I. Hart,
O. E. Hill,
J. F. P. Hodson,
J. Morgan Howe,
Robert Huey,
A. O. Hunt,
Louis Jack,
V. H. Jackson,
C. R. Jefferis,
C. A. Kingsbury,
Edw. C. Kirk,
C. R. E. Koch,
W. T. La Roche,
W. K. Leech,
F. A. Levy,
Wilbur F. Litch,
J. Bond Littig,
Benjamin Lord,
Geo. W. Lovejoy (Canada),
John S. Marshall,
H. J. McKellops,
Charles McManus,
James McManus,
Dan'l Neal McQuillan,
Horatio C. Meriam,
W. D. Miller (Berlin),
W. E. Page,
S. B. Palmer,
C. B. Parker,
D. D. Peabody,
C. N. Peirce,
S. G. Perry,
Chas. E. Pike,
W. A. Phreaner,
W. E. Pinkham,
W. H. Potter,
Chas. P. Pruyn,
H. C. Register,
Z. T. Sailer,
L. D. Shepard,
Eugene H. Smith,
B. Holly Smith,
H. A. Smith,
S. G. Stevens,
W. X. Sudduth,
Eugene S. Talbott,
Ambler Tees,
J. D. Thomas,
James Truman,
Wm. H. Trueman,
W. W. Walker,
I. F. Wardwell,
G. W. Warren,
T. S. Waters,
Geo. W. Weld,
Julius G. W. Werner,
Jacob L. Williams,
R. B. Winder,
C. A. Woodward,
J. A. Woodward,
W. J. Younger.

SUBSCRIBE FOR THE

### INTERNATIONAL DENTAL JOURNAL

FOR 1890.

Its fearless, independent course in defence of Professional interests should make it indispensable to every dentist.

ALL DEALERS ARE OUR AUTHORIZED AGENTS. TERMS, $2.50 PER ANNUM, IN ADVANCE.

